# Identification of Type 2 Diabetes Biomarkers From Mixed Single-Cell Sequencing Data With Feature Selection Methods

**DOI:** 10.3389/fbioe.2022.890901

**Published:** 2022-06-02

**Authors:** Zhandong Li, Xiaoyong Pan, Yu-Dong Cai

**Affiliations:** ^1^ College of Biological and Food Engineering, Jilin Engineering Normal University, Changchun, China; ^2^ Key Laboratory of System Control and Information Processing, Institute of Image Processing and Pattern Recognition, Ministry of Education of China, Shanghai Jiao Tong University, Shanghai, China; ^3^ School of Life Sciences, Shanghai University, Shanghai, China

**Keywords:** type 2 diabetes, single-cell sequencing, Monte Carlo feature selection, support vector machine, RIPPER

## Abstract

Diabetes is the most common disease and a major threat to human health. Type 2 diabetes (T2D) makes up about 90% of all cases. With the development of high-throughput sequencing technologies, more and more fundamental pathogenesis of T2D at genetic and transcriptomic levels has been revealed. The recent single-cell sequencing can further reveal the cellular heterogenicity of complex diseases in an unprecedented way. With the expectation on the molecular essence of T2D across multiple cell types, we investigated the expression profiling of more than 1,600 single cells (949 cells from T2D patients and 651 cells from normal controls) and identified the differential expression profiling and characteristics at the transcriptomics level that can distinguish such two groups of cells at the single-cell level. The expression profile was analyzed by several machine learning algorithms, including Monte Carlo feature selection, support vector machine, and repeated incremental pruning to produce error reduction (RIPPER). On one hand, some T2D-associated genes (MTND4P24, MTND2P28, and LOC100128906) were discovered. On the other hand, we revealed novel potential pathogenic mechanisms in a rule manner. They are induced by newly recognized genes and neglected by traditional bulk sequencing techniques. Particularly, the newly identified T2D genes were shown to follow specific quantitative rules with diabetes prediction potentials, and such rules further indicated several potential functional crosstalks involved in T2D.

## 1 Introduction

Diabetes mellitus (DM) turns out to be the general term describing metabolic disorders with high blood sugar levels as typical symptoms (Tseng et al., 2012; Tao et al., 2015). Due to either lack of insulin or pathogenic insulin reactive responses, diabetes can be divided into three groups: type 1 DM with low insulin production, type 2 DM with insulin resistance, and gestational diabetes with high blood sugar levels induced by diabetes recurrence during pregnancy (American Diabetes Association, 2014). According to the epidemiologic statistics data in 2015, more than four hundred million people suffered from diabetes, and about five million people died from such disease all over the world (Gao et al., 2016; Disease and Injury Incidence and Prevalence Collaborators, 2017; Global Burden of Disease Cancer Collaboration et al., 2017). Particularly, type 2 diabetes (T2D) makes up about 90% of all cases (392 million) and is the primary subtype of diabetes (Disease and Injury Incidence and Prevalence Collaborators, 2017; Global Burden of Disease Cancer Collaboration et al., 2017), indicating such kind of disease is one of the major threats to human health.

Different from type 1 DM and gestational diabetes, the major pathogenesis of type 2 DM is insulin resistance and beta-cell dysfunction accompanied with insufficient insulin secretion (Pandey et al., 2015), where insulin resistance is generally defined as dysfunctional insulin-mediated glucose clearance (Yabe et al., 2015). During the pathogenesis of type 2 DM, the typical insulin-associated biological processes and action cascade are usually disturbed by either intracellular signals or extra interferences, including serine phosphorylation of IRS-1, excess glucosamine, mitochondria defects, FA (fatty acid)-induced insulin dysfunction, and alternate fatty acid effects (Taylor, 2013; Eckardt et al., 2014; Pandey et al., 2015). Early in 1997, Boden (1997) has already demonstrated the significance of fatty acids in diabetes. Further similarly in the same year, functional signaling molecules IRS-1 and IRS-2 were confirmed by Zick (2001), revealing the initial biological foundations for diabetes. Apart from such complicated pathogenesis associated with insulin resistance, beta-cell dysfunction has also been widely identified in type 2 DM patients as the other etiological factor. Similar to insulin resistance, such pathogenesis also has various potential mechanisms, including glucose toxicity, beta-cell exhaustion, impaired proinsulin biosynthesis, and lipo-toxicity (Ferrannini, 2009). In 2003, Kahn (2003) demonstrated the specific contribution of both insulin resistance and beta-cell dysfunction to the pathogenesis of diabetes, laying a foundation for the basic pathological mechanisms of such disease. Different from the downstream mechanisms of two major pathogeneses, such pathogenic mechanisms can be both attributed to either genetic predisposition or environmental interferences (Andersen et al., 2016; Stancakova and Laakso, 2016). They would be involved in the progressive dysfunction of pancreatic islet alpha and beta cells, so that, the pancreatic islet cells actually have specific roles in the initiation and progression of type 2 DM.

Traditionally, the studies on the pathogenic characteristics and contribution of pancreatic islet cells mainly focused on the abnormal biochemical reactions and physiological processes of such cell types in type 2 DM (Borg et al., 2001; Donath et al., 2003; Prentki and Nolan, 2006; Westermark and Westermark, 2008). According to these studies, there are four major pathogenic characteristics of pancreatic islet cells, including increased islet glucose metabolism (Forst et al., 2014), abnormal lipid signaling (Chakraborty et al., 2014), abnormal GLP-1 secretion (Trujillo and Nuffer, 2014), and compensatory feedback stimulation on parasympathetic and sympathetic neurons (Thorens, 2014). With the development of high-throughput sequencing technologies, more and more fundamental pathogenesis of type 2 DM at genetic and transcriptomic levels has been revealed. Apart from such transcription factors, genes regulating optimal glucose-responsive insulin secretion, like *IAPP*, *GLUT2*, *GAD65*, and *IA-2*, have also been identified to participate in T2D-associated pathogenesis (Clocquet et al., 2000). Therefore, the abnormal gene functions of pancreatic islet cells may be one of the major pathogenic factors for type 2 DM. However, as we all know, the cellular components of pancreatic islet cells are quite complicated involving various cell subtypes. Meanwhile, traditional studies all focused on the biological features (either at the cellular level or genetic level) of cell population, no matter pathogenic or not for individual cells. Therefore, these conventional studies may ignore some potential pathogenic factors and mistake non-pathogenic features due to normal or irrelevant cells’ interferences.

Multiple previous studies have focused on single-cell analyses on pancreatic islets under physical or pathological conditions. With the development of single-cell techniques, the studies on pancreatic islets under either pathological or normal conditions have been extended to the single-cell level. Early in 2016, Segerstolpe et al. (2016) have identified some typical biomarkers to distinguish pancreatic islets under healthy and diabetic conditions. However, as limitations of this study, the authors only applied differential expression analyses and the t-SNE method to identify some potential biomarkers to reveal the heterogeneity (Segerstolpe et al., 2016). Apart from this study, further in 2017, another study extended to identify the specific biomarkers for T2D, confirming that genes are differentially expressed at the transcriptomics level not only between patients and controls but also among different cell types (Lawlor et al., 2017). In 2018, another single-cell gene expression analysis on T2D also tried to identify specific biomarkers for the prediction of cellular states of beta-cells, either healthy or T2D beta-cells (Ma and Zheng, 2018). The shortcomings of these two studies turn out to be a lack of quantitative standards establishment, making it still quite hard to predict T2D using single-cell transcriptomics data.

To overcome the limitations of previous studies mentioned earlier, in this study, for the first time, we used the single-cell sequencing results from one previous study (Xin et al., 2016) and tried to extend their analyses at two levels: 1) using multiple machine learning algorithms for deep analysis; 2) taking the pancreatic islets as a whole and did not distinguish different cell subtypes. We extended the classification and prediction of cellular states from just beta cells to multiple cell types, including human pancreatic alpha, beta, delta, and PP cells. Also, different from previous studies, we did not just focus on the pathogenic effects of T2D on beta cells but tried to reveal the general comprehensive pathogenic effects on all the cells from the pancreatic islets. Although most of the previous studies identified that pancreatic islet B cells are the major participants in the pathogenesis of T2D, other cells, including alpha, delta, and PP cells, are also either shown to be correlated with the pathogenesis of T2D or may act as potential biomarkers for T2D due to their typical changes during the pathogenesis. Therefore, it is not only innovative but effective to reveal the comprehensive effects of T2D on pancreatic islets and identify more valuable biomarkers for such disease.

All in all, to remove the interferences caused by conventional bulk sequencing and analysis, we have tried to identify potential pathogenic factors of T2D from the transcriptomic profiling covering multiple cell subtypes at the single-cell level. Relied on single-cell RNA sequencing techniques and related public datasets (Xin et al., 2016), we investigated such datasets with several powerful machine learning algorithms. Different from previous studies, focusing on identifying biomarkers for distinguishing a tissue under normal or pathological conditions but not an entire tissue, which makes hard to detect biomarkers from a single-cell subtype in clinical applications, this study tried to identify the common transcriptomics characteristics across different cell types at the single-cell level for T2D. Biomarkers identified in this study may not be affected by the cell composition of the islet tissue that may vary among different individuals. In addition, our results revealed novel potential pathogenic mechanisms induced by newly recognized genes in a rule manner, which are always neglected by traditional bulk sequencing techniques. On the one hand, these results deepen our understanding on the etiology and pathogenesis of T2D. On the other hand, such identified new biomarkers can be potential candidates for further clinical application in the diagnosis of T2D using the transcriptomics information of the entire tissue, with no further cell separation and preprocessing required.

## 2 Materials and Methods

**FIGURE 1:**
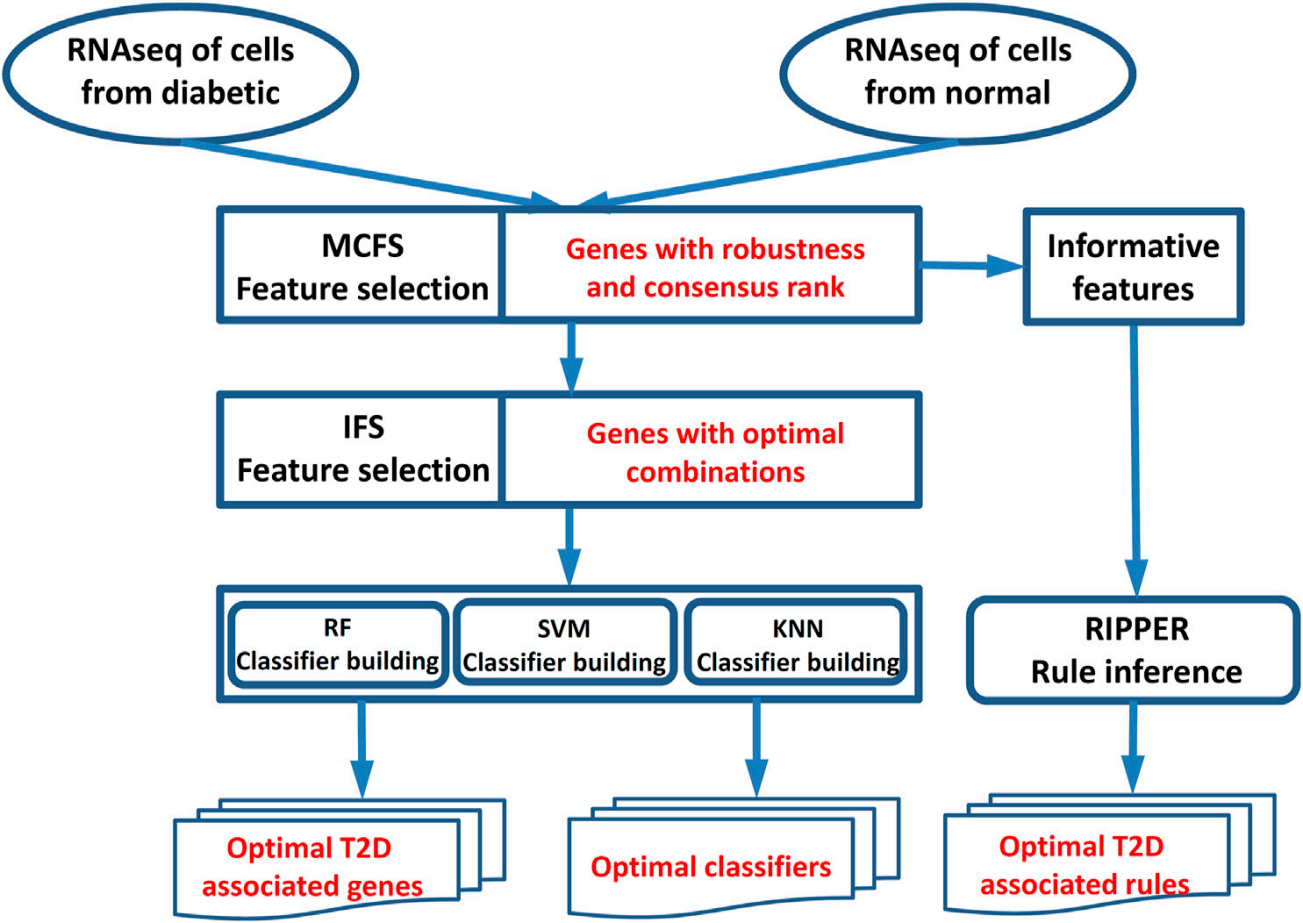
Workflow for key gene identification of type 2 diabetes. The MCFS method was used to evaluate the importance of all features (genes). On the one hand, the IFS method with SVM/RF/KNN was applied on the feature list yielded by the MCFS method to extract optimal T2D-associated genes and optimal classifiers. On the other hand, the informative features yielded by the MCFS method were fed into the Johnson reducer and RIPPER algorithms to construct optimal T2D-associated rules.

In this study, we first used a feature selection method to analyze a RNA sequencing dataset of T2D for ranking the important genes associated with T2D, and these genes were further optimized for diabetes using incremental feature selection (IFS) (Liu and Setiono, 1998) with some supervised classifiers. In the end, we applied the rule learning method to generate interpretable classification rules for T2D. The whole process is illustrated in Figure 1.

### 2.1 Datasets

We downloaded the RNA sequencing data of 1,600 human pancreatic islet cells from the GEO (Transcript Expression Omnibus) database under the accession number of GSE81608 at https://www.ncbi.nlm.nih.gov/geo/query/acc.cgi?acc=GSE81608 (Xin et al., 2016). There were 949 pancreatic islet cells from six T2D patients and 651 pancreatic islet cells from 12 non-diabetic donors. Within the 949 pancreatic islet cells from T2D patients, there were 569 alpha, 296 beta, 30 delta, and 54 PP cells. Within in the 651 pancreatic islet cells from non-diabetic donors, there were 377 alpha, 207 beta, 28 delta, and 39 PP cells. The expression levels of 39,851 genes were quantified as RPKM (Reads Per Kilo bases per Million reads). The processed gene expression profiles of these cells downloaded from https://ftp.ncbi.nlm.nih.gov/geo/series/GSE81nnn/GSE81608/suppl/GSE81608_human_islets_rpkm.txt.gz were used. Despite islet cells containing different cells, this work expects to identify the common gene signatures for T2D across multiple cell types.

### 2.2 Feature Selection

In this study, we first used the Monte Carlo feature selection (MCFS) (Draminski et al., 2008) to evaluate the importance of all genes, obtaining a feature list and some informative genes expressed in diabetes. For the feature list, it was fed into the IFS (Liu and Setiono, 1998) with one classification algorithm to extract optimal genes that had a strong discriminate ability between diabetes and non-diabetes samples and construct an efficient classifier. On the other hand, repeated incremental pruning to produce error reduction (RIPPER) was employed to determine interpretable rules on gene expression patterns with informative features.

#### 2.2.1 Monte Carlo Feature Selection

The investigated data contained 1,600 samples, each of which was represented by expression levels on lots of genes. Accordingly, the data can be summarized as a matrix with low row numbers and high column numbers. MCFS is deemed to be a powerful feature selection method to deal with such data. Thus, it was employed in this study. MCFS is a multivariate feature selection method based on bootstrap samples and decision trees, which focuses on selecting discriminate features for classification with robustness. In this feature selection algorithm, it generates multiple bootstrap sets, and on each bootstrap set, multiple decision trees are grown on smaller feature subsets randomly selected from original features. Then, the involvement of each feature in the decision trees shows a relative importance (RI) score, which indicates the overall number of splits involving this feature in all nodes of all constructed trees. The MCFS program was downloaded from http://www.ipipan.eu/staff/m.draminski/mcfs.html. For convenience, default parameters were adopted.

The MCFS program was executed on the aforementioned RNA sequencing data. According to the output of the MCFS program, we can obtain the RI values of all features. Accordingly, features can be ranked in a list with the decreasing order of their RI values. Furthermore, it also provides the informative features, which are generated by a permutation test on class labels and one-sided Student’s t-test. These features are always the top-ranking features in the list. We would adopt these features to construct classification rules *via* RIPPER.

#### 2.2.2 Incremental Feature Selection

In this study, we performed IFS on the MCFS-generated feature list, denoted by $F=\left[f_{1},f_{2},\ldots ,f_{N}\right]$ (*N* was the total number of features), to screen out a set of optimal features, which can accurately discriminate between diabetes and non-diabetes samples. Based on such list, we generated a series of feature subsets with step 5. Suppose there are *m* feature subsets $\left[F_{1},F_{2},\ldots ,F_{m}\right]$ , where the *i*th feature subset contains top 5×i features, that is, $F_{i}=\left[f_{1},f_{2},\ldots ,f_{i\times 5}\right]$ . Then, for a given classification algorithm, we built one classifier on samples represented by features from each feature subset and yielded the 10-fold cross-validation performance for evaluating this classifier. After all constructed feature subsets had been tested, the feature subset, on which the classifier provided the best performance, can be obtained. Such a feature subset was called the optimal feature subset for this classification algorithm, and the features inside were named as the optimal features. Furthermore, the classifier with the best performance was termed as the optimal classifier.

### 2.3 Classification Algorithm

For the IFS method, one classification algorithm was necessary. In this study, we tried three classic classification algorithms: 1) support vector machine (SVM) (Cortes and Vapnik, 1995), 2) K-nearest neighbor (KNN) (Cover and Hart, 1967), and 3) random forest (RF) (Breiman, 2001). Their brief descriptions were as follows.

#### 2.3.1 Support Vector Machine

The SVM is a supervised learning model based on statistical learning theory and is widely used in many biological problems (Pan and Shen, 2009; Mirza et al., 2015; Chen et al., 2017; Jia et al., 2018; Wei et al., 2018; Zhou et al., 2022a; Zhou et al., 2020b; Liu et al., 2021; Wang et al., 2021; Zhu et al., 2021; Li X. et al., 2022; Wu and Chen, 2022). Given a set of training samples, each training sample is assigned to positives or negatives. The SVM training algorithm fits a hyperplane that has the maximum margin between positives and negatives, where the generalization error becomes smaller when the margin is larger. The SVM generally is good at handling non-linear data, since it can first map the data in non-linear space to high-dimensional linear space by the kernel function and then fit a linear model in the high-dimensional space.

#### 2.3.2 K-Nearest Neighbor

KNN is one of the simplest schemes for classifying samples. However, in many cases, it still can yield good performance. Given a training dataset, KNN directly uses samples in it to make prediction for any query sample, that is, KNN does not contain a learning procedure. Generally, it finds *k* training samples, which have the nearest distances (e.g., Euclidean distance) to the query sample. By counting the classes of these *k* training samples, the class with most votes is assigned to the query sample.

#### 2.3.3 Random Forest

RF is another classic classification algorithm. In fact, it is an integrated algorithm, consisting of several decision trees. For constructing each decision tree, it randomly picks up samples from the training dataset, with replacement, to constitute the basic dataset. The tree is extended at each node by selecting an optimal split on one feature among the randomly selected features. RF integrates the predictions of all decision trees with majority voting. RF is deemed as a powerful classification algorithm and has wide applications in tackling many biological problems (Kandaswamy et al., 2011; Casanova et al., 2014; Marques et al., 2016; Jia et al., 2020; Liang et al., 2020; Zhang et al., 2021b; Chen et al., 2021; Onesime et al., 2021; Chen et al., 2022; Ding et al., 2022; Yang and Chen, 2022).

To quickly implement the aforementioned three classification algorithms, we employed the corresponding packages in scikit-learn (https://scikit-learn.org/stable/). Some main parameters were tuned for extracting optimal parameters.

### 2.4 Johnson Reducer and Repeated Incremental Pruning to Produce Error Reduction Algorithms

Classification algorithms mentioned in Section 2.3 are powerful to construct efficient classifiers. However, we cannot understand their principles because they are black-box algorithms. In this case, few clues for uncovering essential differences between T2D patients and non-diabetic donors can be obtained. In view of this, we further adopted rule learning algorithms to investigate the RNA sequencing data. Although it is generally weaker than the aforementioned algorithms, it can provide rules that clearly indicate special expression patterns on T2D patients, thereby improving our understanding on T2D. The procedures were described in the following sections.

As mentioned in Section 3.2.1, the MCFS method can select some informative features. These features are quite essential to describe the characteristics of the dataset. Here, we used these features to construct classification rules *via* RIPPER algorithm (Cohen, 1995). Before that, the Johnson reducer algorithm (Johnson, 1974) was applied on the informative features to select the most important features, which had the similar classification ability compared to the original informative features. The selected features were fed into the RIPPER algorithm. RIPPER, proposed by Cohen (1995), is a rule learning algorithm which is capable of handling large noisy datasets effectively. RIPPER is the improved version of IREP (Johannes and Widmer, 1994) which combines both the separate-and-conquer technique used first in the relational learner FOIL (Quinlan, 1990) and the reduced error pruning strategy proposed by Brunk and Pazzani (1991). In RIPPER, the training set is first split into growing and pruning sets. Then, repeat the rule grow phase and rule prune phase until no positive samples are left in the growing set, or the description length (DL) is 64 bits greater than the smallest DL found so far, or the error rate is greater than 50%. In the rule grow phase, one rule is generated by greedily adding conditions to the rule that achieves the highest FOIL’s information gain. In the rule prune phase, the rule is pruned using reduced error pruning. Finally, global optimization strategy is applied to further prune the rule set. The aforementioned procedures for constructing rules are also implemented in the MCFS program, that is, the set of rules is one output of the MCFS program.

### 2.5 Performance Measurement

In this study, we used six measurements to evaluate the performance of all classifiers under 10-fold cross-validation (Kohavi, 1995; Li Z. et al., 2022; Ding et al., 2022; Tang and Chen, 2022), including sensitivity (SN) (same as recall), specificity (SP), accuracy (ACC), Matthew correlation coefficient (MCC), precision, and F1-measure (Matthews, 1975; Zhao et al., 2018; Zhao et al., 2019; Jia et al., 2020; Liang et al., 2020; Zhang et al., 2021a; Zhang et al., 2021c; Pan et al., 2021). Their formulations are written as follows:

$$SN=Recall=\frac{TP}{TP+FN},$$ (1)

$$SP=\frac{TN}{TN+FP},$$ (2)

$$ACC=\frac{TP+TN}{TP+TN+FP+FN},$$ (3)

$$MCC=\frac{TP\times TN-FP\times FN}{\sqrt{\left(TP+FP\right)\left(TP+FN\right)\left(TN+FP\right)\left(TN+FN\right)}},$$ (4)

$$Precision=\frac{TP}{TP+FP},$$ (5)

$$F1-measure=\frac{2\times Recall\times Precision}{Recall+Precision},$$ (6)

where TP represents the number of truly positive samples, FP represents the number of false-positive samples, TN represents the number of truly negative samples, and FN represents the number of false-negative samples. Among these six measurements, we selected F1-measure as the key one, whereas others were provided for reference.

### 2.6 Gene Ontology Enrichment Analysis on Optimal Genes

Some rules can be extracted *via* the Johnson reducer and RIPPER algorithms, which involved several features (genes), called rule genes, in the following text. We performed Gene Ontology (GO) enrichment analysis using R package *topGO* (http://bioconductor.org/packages/release/bioc/html/topGO.html, v.2.24.0) on these rule genes. The genes of interest were set as rule genes, and the gene background was set as all the available genes. The *p*-value threshold was set at 0.001.

## 3 Results

T2D is one type of DM and makes up most DM cases. In this study, we investigated potential pathogenic factors of T2D at the single-cell level by analyzing a single-cell RNA sequencing dataset. Such dataset contained 1,600 single cells, including 949 cells from T2D patients and 651 cells from normal controls. It was analyzed by some powerful machine learning algorithms, including MCFS (Draminski et al., 2008), SVM (Cortes and Vapnik, 1995), KNN (Cover and Hart, 1967), RF (Breiman, 2001), and RIPPER (Cohen, 1995). The entire procedure is shown in Figure 1. On one hand, we obtained some T2D-associated genes, which can be novel biomarkers of T2D. On the other hand, some interesting rules were constructed, which can uncover different expression patterns in T2D patients and normal controls. This section gives the detailed results of these procedures.

## 3.1 Results of the Monte Carlo Feature Selection Method

The MCFS method was directly applied to the RNA sequencing data to analyze the importance of all features (genes). Each gene was assigned a RI score. A total of 26,978 genes were assigned RI scores larger than zero. These genes and their RI scores are provided in Supplementary Table S1. Because the RI scores of the rest genes were zero, meaning their associations for the identification of T2D samples were very weak, they were discarded. A feature list was generated by sorting the remaining 26,978 genes in the decreasing order of their RI scores, which is also provided in Supplementary Table S1.

In addition to the feature list, the MCFS method can output some informative features. For investigating RNA sequencing data, 235 informative features were extracted by the MCFS method, which were the top 235 genes listed in Supplementary Table S1.

## 3.2 Results of the Incremental Feature Selection Method

To further extract optimal features, the IFS method combined with one classification algorithm was employed. Here, we tried three classification algorithms: SVM, KNN, and RF. Some main parameters of each algorithm were tuned. In detail, for SVM, four kernels were attempted, including linear, polynomial, RBF, and sigmoid kernels. The parameter *k* for KNN was set to 1, 5, and 10, and the parameter, number of decision trees (I), for RF was set to 20, 40, 60, 80, and 100. Because the feature list contained a huge number of features, we only considered the top 5,000 features in this study to save time. Several feature subsets were constructed using step 5.

**FIGURE 2:**
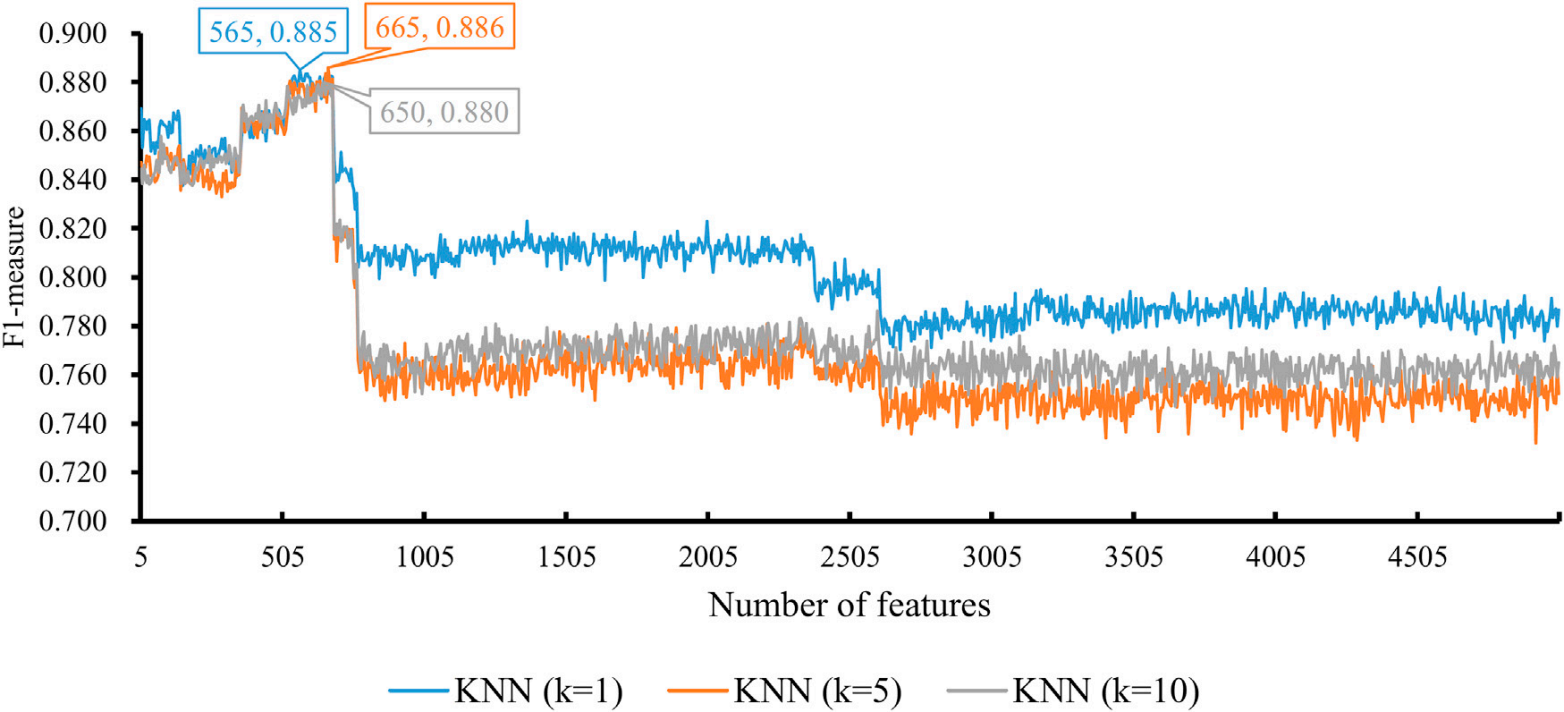
Performance of KNN integrated in IFS using different numbers of features. The y-axis is F1-measure, and the x-axis is the number of participated features. *k* is the parameter of KNN, indicating the number of nearest neighbors that are used to make prediction. KNN can yield the best F1-measure of 0.886 when *k* = 5 and the top 665 features are used.

**FIGURE 3:**
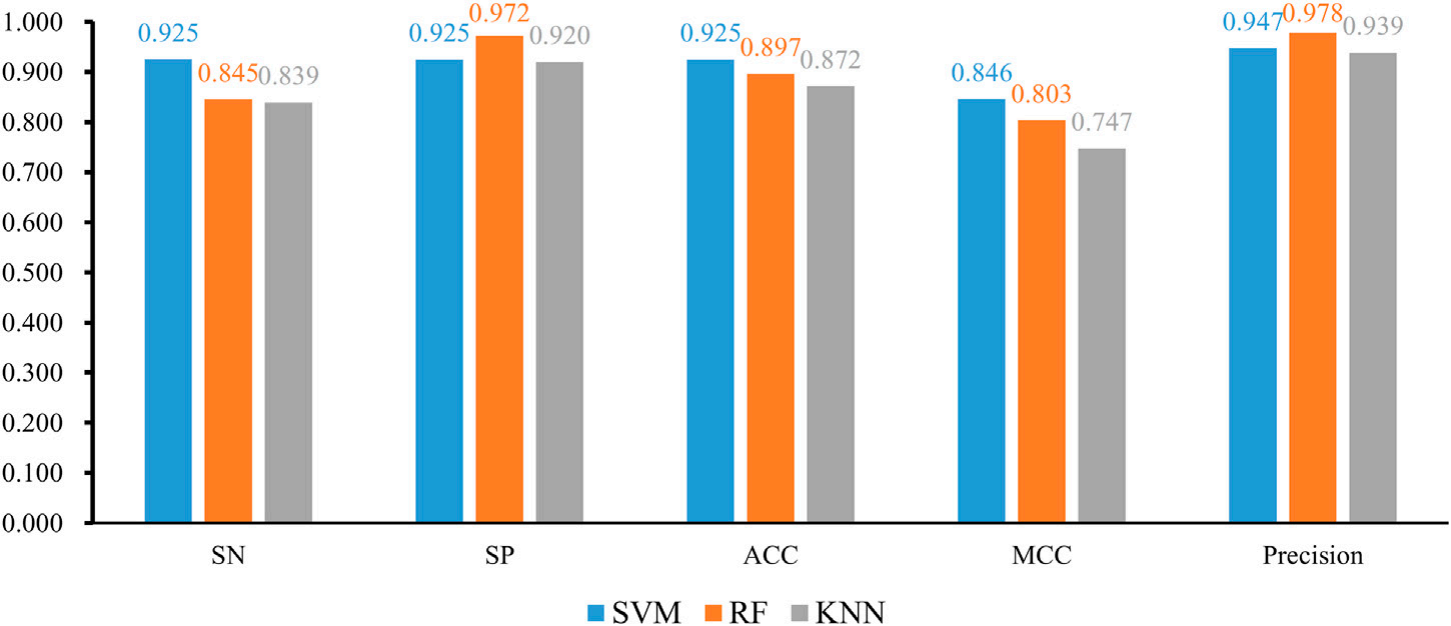
Bar chart to show five measurements of three optimal classifiers based on different classification algorithms.

When the classification algorithm was KNN, several KNN classifiers with a certain parameter *k* were constructed on all feature subsets. All these classifiers were evaluated by 10-fold cross-validation. The obtained six measurements are listed in Supplementary Table S2. For an easy observation, we plot a curve for KNN with a certain parameter *k*, as shown in Figure 2, in which the F1-measure was set to the y-axis and the number of features was set to the x-axis. We can see that when *k* = 1, 5, and 10, the highest F1-measure was 0.885, 0.886, and 0.880, respectively. Thus, the KNN classifier with *k* = 5 provided the best performance. Such classifier used the top 665 features (genes) in the feature list. These features were the optimal features for KNN. The other five measurements are illustrated in Figure 3. Except MCC, all measurements exceeded 0.8, implying the good performance of such KNN classifiers. Furthermore, it can be observed from Figure 2 that the IFS curves of KNN with different parameters *k* had a common feature. The curve followed a sharp decreasing trend before about top 600 features were used. The top features in the list were highly related to class labels (T2D patients and non-diabetic patients in this study), and a simple scheme based on these features, as KNN used, can correctly predict the cells of T2D patients and non-diabetic patients. However, when features with low ranks, which had low relevance to class labels, were added, KNN cannot exclude interference information contained in these features as KNN has no training procedures, inducing the quick descent of its performance. In this study, the set containing about top 600 features was a pivotal point for KNN. After this point, the performance of KNN followed a sharp decreasing trend.

**FIGURE 4:**
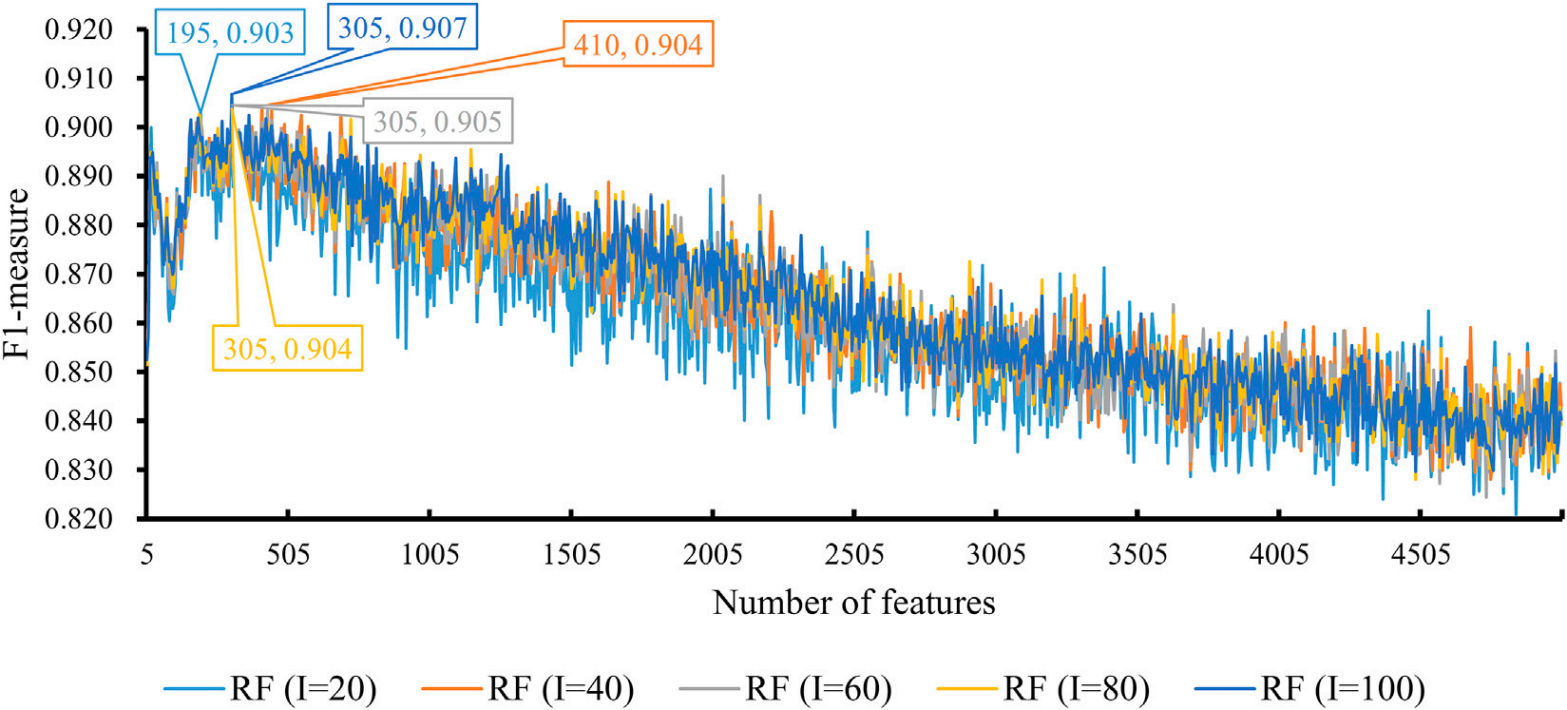
Performance of RF integrated in IFS using different numbers of features. The y-axis is F1-measure, and the x-axis is the number of participated features. *I* is the parameter of RF, indicating the number of decision trees. RF can yield the best F1-measure of 0.907 when *I* = 100 and the top 305 features are used.

We also tried another classification algorithm, RF. The same IFS procedure was conducted on this algorithm. The obtained measurements are listed in Supplementary Table S3. Likewise, a curve was plotted for RF with a certain number of decision trees, as shown in Figure 4. It can be observed that when *I* = 20, 40, 60, 80, and 100, the highest F1-measure was 0.903, 0.904, 0.905, 0.904, and 0.907. The RF classifier with *I* = 100 provided the highest performance. The top 305 features in the list were adopted in this classifier and were termed as optimal features for RF. Evidently, such an RF classifier was superior to the best KNN classifiers mentioned earlier. Furthermore, the other five measurements of this RF classifier are shown in Figure 3. All measurements were higher than 0.8, suggesting the better performance of this classifier than the aforementioned KNN classifier.

**FIGURE 5:**
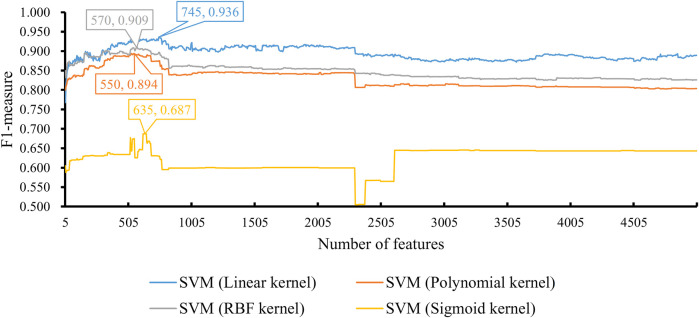
Performance of SVM integrated in IFS using different numbers of features. The y-axis is F1-measure, and the x-axis is the number of participated features. SVM can yield the best F1-measure of 0.936 when the kernel is a linear function and the top 745 features are used.

Finally, we conducted the same IFS procedure for SVM. The measurements are listed in Supplementary Table S4. Similarly, for each SVM with a certain kernel, a curve was plotted, as shown in Figure 5. With four different kernels, SVM yielded the highest F1-measure of 0.936, 0.894, 0.909, and 0.687. The SVM with a linear kernel provided the best performance. Also, such performances were based on the top 745 features in the list. Accordingly, they were called the optimal features for SVM. Furthermore, the performance of this SVM classifier was better than that of the aforementioned KNN and RF classifiers. The same conclusion can be obtained according to the five measurements of such SVM classifiers, illustrated in Figure 3. Due to the best performance of the SVM with its optimal 745 genes, these genes were quite important for investigating T2D at the single-cell level. The top seven genes are listed in Table 1.

**TABLE 1:** Top seven genes among the optimal genes for SVM.

Rank	Gene ID	Gene symbol	RI
1	100128906	LOC100128906	0.1140
2	100873254	MTND4P24	0.1046
3	100271063	RPS14P1	0.1032
4	100652939	MTND2P28	0.0979
5	285045	LINC00486	0.0959
6	729898	ZBTB8OSP2	0.0954
7	391524	THRAP3P1	0.0862

With the earlier IFS results with different classification algorithms using various parameters, the SVM with linear kernel and top 745 features provided the best performance of F1-measure 0.936. The ACC and MCC of such classifier were 0.925 and 0.846, respectively. Other three measurements, SN, SP, and precision were 0.925, 0.925, and 0.947, respectively. These measurements suggested the excellent performance of this classifier, and it can be an efficient tool to identify cells of T2D patients.

### 3.3 Classification Rules

Although we can construct efficient classifiers to identify cells of T2D patients through three classification algorithms, these classifiers were absolute black-box algorithms, which prevented us from uncovering the essential differences between cells of T2D patients and non-diabetic donors. As mentioned in Section 2.4, rule learning algorithms were employed.

According to the output of the MCFS program, 235 features were selected as informative features. To test the utility of the classification rules yielded by Johnson reducer and RIPPER algorithms, we performed the 10-fold cross-validations three times, obtaining the F1-measure of 0.910, which was lower than that of the optimal SVM classifier but higher than that of the optimal KNN and RF classifiers. The SN was 0.898, SP was 0.891, ACC was 0.895, MCC was 0.784, and precision was 0.923. Although such performance was lower than that of the optimal SVM classifier, the RIPPER algorithm can construct a group of rules, which made the classification procedure completely open and provided more insights. Thus, the Johnson reducer and RIPPER algorithms were applied to all samples, producing nine different classification rules, as listed in Table 2. These rules are able to accurately screen patients with T2D from non-diabetic population. Although these rules were mainly for non-diabetes, based on the aforementioned evaluation results (SP = 0.891), it was believed that these rules were statistically shown to cover almost all possible non-diabetes samples. Thus, investigation on these rules can also figure out the characteristics of T2D patients in an opposite aspect.

**TABLE 2:** Nine classification rules for diabetes generated by the RIPPER algorithm.

Rule	Criteria	Patient
Rule 1	Gene Id 100128906 (LOC100128906) ≥ 2.7722	Non-diabetes
	Gene Id 326307 (RPL3P4) ≤ 15.2306	
	Gene Id 8781 (PSPHP1) ≥ 0.0965	
	Gene Id 100873065 (PTCHD1-AS) ≤ 0.1036	
Rule 2	Gene Id 100462954 (MICOS10P3) ≥ 2.0984	Non-diabetes
	Gene Id 1487 (CTBP1) ≤ 17.3460	
	Gene Id 326307 (RPL3P4) ≤ 6.2868	
	Gene Id 100873254 (MTND4P24) ≥ 3.0364	
Rule 3	Gene Id 100128906 (LOC100128906) ≥ 49.6340	Non-diabetes
	Gene Id 143244 (EIF5AL1) ≥ 1.0987	
	Gene Id 486 (FXYD2) ≤ 152.8666	
	Gene Id 326307 (RPL3P4) ≤ 11.3894	
	Gene Id 6126 (RPL9P7) ≤ 103.5050
Rule 4	Gene Id 100128906 (LOC100128906) ≥ 3.0256	Non-diabetes
	Gene Id 326307 (RPL3P4) ≤ 22.4381	
	Gene Id 100128906 (LOC100128906) ≥ 225.8732	
	Gene Id 388147 (RPL9P9) ≤ 50.3934	
	Gene Id 100271332 (RPL36AP21) ≥ 1.7952	
	Gene Id 222901 (RPL23P8) ≤ 2.6067	
Rule 5	Gene Id 100652939 (MTND2P28) ≥ 450.8125	Non-diabetes
	Gene Id 4574 (MT-TS1) ≤ 445.4115	
	Gene Id 1487 (CTBP1) ≤ 37.6438	
Rule 6	Gene Id 285045 (LINC00486) ≤ 0.0930	Non-diabetes
	Gene Id 100873254 (MTND4P24) ≤ 28.2479	
	Gene Id 653147 (RPL26P30) ≥ 5.1856	
	Gene Id 285900 (RPL6P20) ≥ 0.4760	
	Gene Id 643932 (RPS3AP20) ≥ 5.5063	
Rule 7	Gene Id 100128906 (LOC100128906) ≥ 3.0256	Non-diabetes
	Gene Id 440737 (RPL35P1) ≥ 4.118	
	Gene Id 100271003 (RPL34P18) ≥ 9.0166	
Rule 8	Gene Id 100128906 (LOC100128906) ≥ 109.2232	Non-diabetes
	Gene Id 100873254 (MTND4P24) ≤ 28.3353	
	Gene Id 644972 (RPS3AP26) ≥ 53.5552	
	Gene Id 644604 (EEF1A1P12) ≤ 7.9556	
Rule 9	Others	Diabetes

### 3.4 Comparison of Classifiers With Informative Features

The MCFS method can directly output some informative features. These features can capture essential information of the dataset. Here, as mentioned in Section 3.3, 235 features were selected as informative features. We can directly use them to construct classifiers with different classification algorithms. These classifiers were also evaluated by 10-fold cross-validation. The main measurement, F1-measure, of these classifiers is listed in Table 3. For KNN, F1-measure varied between 0.839 and 0.849. The F1-measure of RF changed between 0.889 and 0.897. Also, SVM provided the F1-measure varying between 0.631 and 0.886. Compared with the F1-measure yielded by the optimal classifier based on the corresponding classification algorithm, the classifier using informative features generated a lower F1-measure, suggesting that such a classifier was inferior to the optimal classifier. The employment of the IFS method can help us construct more efficient classifiers.

**TABLE 3:** Performance of classifiers using informative features yielded by the MCFS method.

Classification algorithm	F1-measure	Decrement^a^
KNN (k = 1)	0.849	0.036
KNN (k = 5)	0.839	0.047
KNN (k = 10)	0.847	0.033
RF (I = 20)	0.889	0.014
RF (I = 40)	0.891	0.013
RF (I = 60)	0.894	0.011
RF (I = 80)	0.894	0.010
RF (I = 100)	0.897	0.010
SVM (linear kernel)	0.882	0.054
SVM (polynomial kernel)	0.859	0.035
SVM (RBF kernel)	0.886	0.023
SVM (sigmoid kernel)	0.631	0.056

a Numbers listed in this column indicate the difference of F1-measure yielded by the optimal classifier and that listed in the second column of this table.

## 4 Discussion

As we have described earlier, we applied our newly presented computational framework to the expression profiling data of more than 1,600 single pancreatic islet cells, constituting 949 diabetic cells and 651 non-diabetic cells (Xin et al., 2016). Based on such a bioinformatics approach, we not only screened out a group of discriminative genes that have distinctive expression patterns in diabetic or non-diabetic cells but also set up a series of quantitative rules for the recognition of pathogenic cells at the single-cell level. According to recent literature reports, several identified genes and established rules could be validated by existing experimental datasets, indicating the efficacy and accuracy of our analysis. The detailed functional analysis and evaluation of each predicted genes with high informative rank and their optimal rules in the expression pattern have been summarized and introduced in the following sections.

### 4.1 Analysis of Optimal Type 2 Diabetes-Associated Genes

Because the optimal SVM classifier provided the best performance, which used top 745 features (genes), we focused on these 745 genes. However, it is impossible to analyze them one by one. Here, only top seven genes were analyzed, which are listed in Table 1.

The first predicted gene, *WDR45-like pseudogene* (100128906), is the pseudogene of gene *WDR45*. According to recent publications, it encodes a functional lncRNA associated with the regulation of *WDR45* (Tsuyuki et al., 2014; Lebovitz et al., 2015). *WDR4*5 has been functionally related to autophagy (Lebovitz et al., 2015). Considering that abnormal autophagy has been well known to contribute to the pathogenesis of T2D (Lee, 2014), it is reasonable to speculate that the expression level of *WDR45* and its upstream regulator (i.e., our predicted gene *LOC100128906*) may have quite different expressions in diabetic pancreatic islets cells compared to normal cells.

The next identified gene is *MTND4P24* (100873254), which is shown to have quite different expression levels in diabetic and normal tissues containing multiple cell subtypes. As an lncRNA-encoding pseudogene, the expression level of such a gene is able to reflect the regulatory ability of lncRNAs on its target gene, *MT-ND4* (Torrell et al., 2013; Mella et al., 2016). Recent publications also confirmed that the expression level of the target gene *MT-ND4* is functionally related to cellular insulin sensitivity in rat models (Houstek et al., 2012). Therefore, as one regulator of *MT-ND*4’s expression, the expression pattern of *MTND4P24* may involve in the pathogenic insulin sensitivity decreasing in type 2 diabetic cells. Similarly, a homolog of *MTND4P24* and *MTND2P28* (100652939) has also been predicted to have different expression levels in multiple cell subtypes from pathogenic or normal pancreatic islets. Considering its similar regulatory mechanisms and the biological function of MTND2, it is also quite convincing to regard such a gene as a potential distinctive standard for diabetic and non-diabetic cells (Mathews et al., 2005).

The predicted gene, *RPS14P1* (100271063), is also a pseudogene, contributing to the regulation of ribosomal protein S14’s expression (Aubert et al., 1992). Meanwhile, the function of ribosomal protein S14 is widely reported to participate in p53-dependent cell-cycle arrest by interacting with *MDM2* (Zhou et al., 2013), which is abnormally activated during the pathogenesis of diabetes (Golubnitschaja et al., 2006; Garufi et al., 2017). Thus, it is a reasonable assumption that ribosomal protein S14 together with *RPS14P1* has different expression levels in normal and diabetic cells.

Apart from such predicted pseudogenes, we also identified some functional lncRNAs that may have different expression patterns in normal and diabetic cells. LINC00486 (285045) is a predicted lncRNA that contributes to the distinction of normal and diabetic cells. According to recent publications, various functional lncRNAs (Liu et al., 2014; Pullen and Rutter, 2014), including LINC00486, have been confirmed to contribute to the initiation and progression of T2D (Pullen and Rutter, 2014).

The following predicted gene, named *ZBTB8OSP2* (729898), is a pseudogene and has been reported to contribute to anti-saccade response and eating disorders (Cornelis et al., 2014; Broer and van Duijn, 2015). As a transcriptional regulator for *ZBTB8*, such genes may indirectly contribute to a specific complication of T2D, the refractory diabetes insipidus, especially in adolescent male patients (Soto et al., 2014). Therefore, we can infer that such genes together with their downstream binding targets may have respective specific expression patterns in normal and diabetic cells.

The next predicted gene is *THRAP3P1* (391524), the pseudogene of *THRAP3*. The post-transcriptional regulatory target of *THRAP3* has been confirmed to dock on phosphoserine 273 of PPAR-gamma and further contribute to the pathogenic programming of diabetic genes, inducing insulin resistance (Choi et al., 2014). Therefore, to accomplish the regulatory role, such gene has a high expression level in normal cells compared to diabetic cells.

### 4.2 Specific Role of Pseudogenes in Type 2 Diabetes-Associated Genes

As we have discussed earlier, we identified multiple pseudogenes associated with T2D. Pseudogenes are nonfunctional segments with similar or reverse sequences of actual coding genes. The biological functions of pseudogenes are still unclear. It has only been speculated that pseudogenes participate in the post-transcriptional regulation *via* generating siRNAs, piRNAs, microRNAs, or other small RNAs (Guo et al., 2009). Although pseudogenes cannot generate protein products, the regulatory effects of such group of genes may still be significant under physical and pathological conditions (Tay et al., 2014). For transcriptomics analyses, especially for single-cell transcriptomics analyses, multiple pseudogenes have been identified as candidate biomarkers for different systematic diseases in studies specifically focusing on pseudogenes’ effects (Kalyana-Sundaram et al., 2012; Poliseno et al., 2015). For most previous studies, the pseudogenes were removed in the data preprocessing. Therefore, most previous studies have not identified a lot of pseudogenes as potential candidate biomarkers for diabetes. In our study, we did not filter out the pseudogenes and for the first time confirmed that pseudogenes with potential transcriptomic regulatory effects may further contribute to the regulation of specific diseases *via* regulating the biological functions of their respective recognized protein-coding genes.

### 4.3 Comparison With Previously Reported Type 2 Diabetes Biomarkers

Here, in this study from other perspective of view, we applied several machine learning algorithms to identify new potential biomarkers for T2D patients. Multiple previous publications have already identified a group of T2D biomarkers such as HbA1c, advanced glycation end-products (AGEs), and pigment epithelial-derived factor (PEDF) (Lyons and Basu, 2012). Also, for the publication from which we retrieved the single-cell sequencing data, unique biomarkers like *LINC00486*, *ZNF445*, and *SYBU* have also been identified for T2D (Xin et al., 2016). Compared with these prediction results, first, we identified a group of confirmed biomarkers like *LINC00486*, validating the efficacy and accuracy of our results. Second, we identified a group of new biomarkers like *MTND4P24* and *THRAP3P1*. Although such genes have been shown to be functionally correlated with T2D, previous studies have not identified such genes as potential biomarkers of T2D. There are two major advantages of our studies compared to previous studies, which may lead us to find novel biomarkers:

1) First, compared with previous studies, we used the single-cell level data with the gene expression profiling of different cells and not just an averaged comprehensive value for each patient. Therefore, we can identify potential biomarkers that are missing due to the averaging procedures.
2) Second, due to the sample size and cell type distribution, it is not proper to use feature selection and machine learning models for distinguishing each cell type independently. An integration of all the cell types may lead to a more reasonable result with effective biomarkers with clinical application potentials.

Such advantages explained why we identified novel protein biomarkers to distinguish T2D patients from normal controls. As we have discussed earlier, some identified biomarkers have been functionally correlated with T2D, implying that it is reasonable to regard such genes/transcripts as potential biomarkers for T2D.

### 4.4 Analysis of Optimal Type 2 Diabetes-Associated Rules

We also screened out a group of functional quantitative rules of the gene expression pattern to distinguish non-diabetic cells from diabetic ones with more interpretability, which are listed in Table 2. Many qualitative rules can be validated according to the gene expression level in existing databases and recent reports on gene expression trends, which support the efficacy and accuracy of the rules. The detailed analysis of each expression rule is widely discussed as follows:

The first rule (rule1) involved four genes including *LOC100128906* [(100128906), *RPL3P4* (326307), *PSPHP1* 8781], and *PTCHD1* (100873065). As mentioned earlier, gene *LOC100128906* has been reported to have quite different transcriptomics patterns between normal and diabetic cells, inhibiting autophagy (Lebovitz et al., 2015). As the antagonistic gene of diabetes-associated autophagy, such genes are reasonable to have high expression in normal cells compared to diabetic cells. As for gene *RPL3P4*, the regulatory target of such pseudogene, *RPL3* has been reported to have a quite low expression level in diabetic cells compared to normal cells (Tsai et al., 1994), corresponding with this rule. As for *PSPHP1* (8781), it has been shown to be associated with the macrophage-related inflammation processes (Walker et al., 2015). Considering that during the initiation and progression of diabetes, regional and systematic inflammation have been widely observed (Donath et al., 2003; Lontchi-Yimagou et al., 2013), it is reasonable to predict such genes as quantitative parameters for the distinction of non-diabetes and diabetes. As for *PTCHD1*, although no direct evidence confirms its contribution on diabetes, it has been confirmed that such a gene is associated with the eye and ear complications of diabetes (Gambin et al., 2017), consistent with this rule.

As for the second rule (rule2), four genes were involved including *MICOS10P3* (100462954), *CTBP1* (1487), *RPL3P4* (326307), and *MTND4P24* (100873254). Few publications have reported the biological contribution of *MICOS10P3*; therefore, it is hard to interpret such gene’s contribution on T2D. As for gene *CTBP1*, it has been reported to participate in the abnormal phosphorylation processes (Kim et al., 2013) and shows a quite high expression level in diabetic cells compared to normal controls. As for gene *RPL3P4*, the regulatory target of such a pseudogene, *RPL3* has been reported to have a quite low expression level in diabetic cells compared to normal cells (Tsai et al., 1994), corresponding with such a rule. *MTND4P24* and its homolog, *MTND5P11*, have been confirmed to regulate a group of functional mitochondrial-encoded NADH ubiquinone oxidoreductase. According to recent publications, during the pathogenesis of diabetes, *MT-ND4* has a quite low-expression pattern and on the contrary, *MT-ND5* has a relevantly higher expression level, corresponding with the prediction expression level of their agonists individually (Elango et al., 2014; Urbanova et al., 2017).

In the third rule (rule3), apart from genes we have discussed earlier, the gene *EIF5AL1* (143244) has also been predicted to have a higher expression pattern in normal cells but not in diabetic cells. Considering the abnormal endocrine stress responses of diabetic cells (Siddiqui et al., 2015), the lower expression level of *EIF5AL1* may also contribute to the identification of diabetic cells. *FXYD2* (486) has been shown to contribute to the pathogenesis of diabetes (Ding et al., 2019). Another specific gene in rule3 is the homolog of *RPL3P4*, *RPL9P7*, which may also participate in the regulation of the pathogenesis of T2D with similar expression patterns to *RPL3P4*.

From the fourth to eighth rules, most of the involved genes occurred in the top three rules or were the top T2D-associated genes just with different combination patterns. Specific genes, like *RPL9P9* (388147) and *RPL36AP21* (100271332) for rule4, *MT-TS1* (4574) for rule5, *RPL26P30* (653147) and *RPL6P20* (285900) for rule6, *RPL35P1* (440737) for rule7, and *RPS3AP26* (644972) for rule8, have been identified in our quantitative rules. As we can see from such typical rule associating biomarkers, most of the genes are ribosome-associated genes like *RPL3P4* (326307) as discussed earlier. Although no direct evidence confirmed the associations between such genes and T2D, it is still reasonable to speculate that such genes may play an irreplaceable role in the identification of T2D. As for *MT-TS1*, such genes have already been reported as potential biomarkers for T2D (Mannino and Sesti, 2012), corresponding with our prediction.

### 4.5 Potential Applications of Identified Type 2 Diabetes-Associated Genes and Rules

There are two potential applications for identified T2D-associated genes: 1) potential biomarkers for T2D diagnosis and monitoring; 2) potential drug target for T2D therapy.

For the identified T2D-associated genes, considering that such genes are identified from pancreatic tissues, they can reflect the original tissue alterations during T2D initiation and progression. Therefore, such genes can be used as biomarkers for direct pancreatic islet biopsy examinations. Apart from that, the candidate genes as potential drug targets can also be manually regulated to prevent the initiation and progression of T2D. Using high-throughput drug screening, antibodies or chemicals specifically targeting the candidate genes can be identified and developed as potential target drugs for T2D.

For the quantitative T2D-associated rules, although we have already identified a group of genes associated with T2D, it is still quite difficult to diagnose T2D. With specific quantitative rules, the identification of T2D patients can be more accurate and efficient. Also, the rules can also be summarized as clinical guidelines for T2D diagnosis using pancreatic tissue single-cell sequencing techniques.

### 4.6 Functional Interpretation of Significant Rule Genes

As listed in Table 2, we identified quantitative rules associated with T2D. The GO enrichment analyses on rule genes were conducted. Table 4 lists the enriched GO terms of these rule genes. It was indicated that most rules are shown to be associated with ribosome-associated biological processes. According to recent publications, ribosome-associated biological processes have been widely shown to be associated with the pathogenesis of T2D. In 2019, in a metabolic study on pancreatic tissues, ribosome-associated genes have been shown to participate in the ERK/hnRNPK/DDX3X pathway in pancreatic islet cells and further regulated the initiation and progression of T2D (Good et al., 2019), consistent with our results. Apart from that, in 2020, DIMT1, as a regulator of ribosomal biogenesis has been shown to participate in the physical biological processes of pancreatic tissue, further validating our results.

**TABLE 4:** Significant Gene Ontology enrichment analysis result on rule genes.

GO ID	Term	*p*-value	Cluster
GO:1903408	Positive regulation of sodium: potassium-exchanging ATPase activity	5.30E-04	BP
GO:0045901	Positive regulation of translational elongation	7.00E-04	BP
GO:0045905	Positive regulation of translational termination	7.00E-04	BP

### 4.7 Limitations of Current Analyses

In this study, for the first time, we adopted several machine learning algorithms to identify disease-specific biomarkers using the mixed single-cell sequencing data. Such analyses may not only identify biomarkers from the single-cell level, getting rid of the bias generated by the averaged transcriptomics using the bulk sequencing method, but also overcome the sample size restriction of traditional single-cell analysis. Compared with traditional single-cell analysis, we did not focus on the classification of different cell subtypes but just the patients and control subjects, improving the analysis accuracy. However, there still remain three major limitations of current analyses on pancreatic single-cell sequencing data:

1) First, the dataset we used is still a relatively small dataset, with around 20 subjects. A larger single-cell sequencing dataset may improve the efficacy and accuracy of our results.
2) Second, the number of cells in each group is not balanced in the raw dataset. Although in the original publications the authors have claimed that the sampling procedure does not affect the distribution of cell subgroups in each subject, a more balanced dataset may perform better.
3) Single-cell sequencing always misses a lot of genes at low-expression levels which cannot be detected at the single-cell level but can be identified in bulk sequencing. Our analyses may also lose the gene expression profiling and analysis on such low-expression genes.

## Data Availability

Publicly available datasets were analyzed in this study. These data can be found at: https://www.ncbi.nlm.nih.gov/geo/query/acc.cgi?acc=GSE81608.

## References

[B1] American Diabetes Association (2014). Diagnosis and Classification of Diabetes Mellitus. Diabetes Care 37 (Suppl. 1), S81–S90. 10.2337/dc14-S081 24357215

[B2] Andersen M. K. Pedersen C.-E. T. Moltke I. Hansen T. Albrechtsen A. Grarup N. (2016). Genetics of Type 2 Diabetes: the Power of Isolated Populations. Curr. Diab Rep. 16, 65. 10.1007/s11892-016-0757-z 27189761

[B3] Aubert D. Bisanz-Seyer C. Herzog M. (1992). Mitochondrial Rps14 Is a Transcribed and Edited Pseudogene in *Arabidopsis thaliana* . Plant Mol. Biol. 20, 1169–1174. 10.1007/bf00028903 1463850

[B4] Boden G. (1997). Role of Fatty Acids in the Pathogenesis of Insulin Resistance and NIDDM. Diabetes 46, 3–10. 10.2337/diabetes.46.1.3 8971073

[B5] Borg H. Gottsäter A. Landin-Olsson M. Fernlund P. Sundkvist G. (2001). High Levels of Antigen-specific Islet Antibodies Predict Futureβ -Cell Failure in Patients with Onset of Diabetes in Adult Age1. J. Clin. Endocrinol. Metabolism 86, 3032–3038. 10.1210/jcem.86.7.7658 11443164

[B6] Breiman L. (2001). Random Forests. Mach. Learn. 45, 5–32. 10.1023/a:1010933404324

[B7] Broer L. Van Duijn C. M. (2015). GWAS and Meta-Analysis in Aging/Longevity. Adv. Exp. Med. Biol. 847, 107–125. 10.1007/978-1-4939-2404-2_5 25916588

[B8] Brunk C. A. Pazzani M. J. (1991). “An Investigation of Noise-Tolerant Relational Concept Learning Algorithms,” in Proceedings of the Eighth International Conference, Evanston, Illinois, June, 1991, 389–393. 10.1016/b978-1-55860-200-7.50080-5

[B9] Casanova R. Saldana S. Chew E. Y. Danis R. P. Greven C. M. Ambrosius W. T. (2014). Application of Random Forests Methods to Diabetic Retinopathy Classification Analyses. PLoS One 9, e98587. 10.1371/journal.pone.0098587 24940623 PMC4062420

[B10] Chakraborty C. Doss C. G. P. Bandyopadhyay S. Agoramoorthy G. (2014). Influence of miRNA in Insulin Signaling Pathway and Insulin Resistance: Micro-molecules with a Major Role in Type-2 Diabetes. WIREs RNA 5, 697–712. 10.1002/wrna.1240 24944010

[B11] Chen L. Li Z. Zhang S. Zhang Y. H. Huang T. Cai Y. D. (2022). Predicting RNA 5-methylcytosine Sites by Using Essential Sequence Features and Distributions. Biomed. Res. Int. 2022, 4035462. 10.1155/2022/4035462 35071593 PMC8776474

[B12] Chen L. Wang S. Zhang Y.-H. Li J. Xing Z.-H. Yang J. (2017). Identify Key Sequence Features to Improve CRISPR sgRNA Efficacy. IEEE Access 5, 26582–26590. 10.1109/access.2017.2775703

[B13] Chen W. Chen L. Dai Q. (2021). iMPT-FDNPL: Identification of Membrane Protein Types with Functional Domains and a Natural Language Processing Approach. Comput. Math. Methods Med. 2021, 7681497. 10.1155/2021/7681497 34671418 PMC8523280

[B14] Choi J. H. Choi S.-S. Kim E. S. Jedrychowski M. P. Yang Y. R. Jang H.-J. (2014). Thrap3 Docks on Phosphoserine 273 of PPARγ and Controls Diabetic Gene Programming. Genes Dev. 28, 2361–2369. 10.1101/gad.249367.114 25316675 PMC4215181

[B15] Clocquet A. R. Egan J. M. Stoffers D. A. Muller D. C. Wideman L. Chin G. A. (2000). Impaired Insulin Secretion and Increased Insulin Sensitivity in Familial Maturity-Onset Diabetes of the Young 4 (Insulin Promoter Factor 1 Gene). Diabetes 49, 1856–1864. 10.2337/diabetes.49.11.1856 11078452

[B16] Cohen W. W. (1995). “Fast Effective Rule Induction,” in Proceedings of the Twelfth International Conference on Machine Learning, Tahoe City, CA, July 9–July 12, 1995, 115–123. 10.1016/b978-1-55860-377-6.50023-2

[B17] Cornelis M. C. Rimm E. B. Curhan G. C. Kraft P. Hunter D. J. Hu F. B. (2014). Obesity Susceptibility Loci and Uncontrolled Eating, Emotional Eating and Cognitive Restraint Behaviors in Men and Women. Obesity 22, E135–E141. 10.1002/oby.20592 23929626 PMC3858422

[B18] Cortes C. Vapnik V. (1995). Support-vector Networks. Mach. Learn 20, 273–297. 10.1007/bf00994018

[B19] Cover T. Hart P. (1967). Nearest Neighbor Pattern Classification. IEEE Trans. Inf. Theory 13, 21–27. 10.1109/tit.1967.1053964

[B20] Ding L. Fan L. Xu X. Fu J. Xue Y. (2019). Identification of Core Genes and Pathways in Type 2 Diabetes Mellitus by Bioinformatics Analysis. Mol. Med. Rep. 20, 2597–2608. 10.3892/mmr.2019.10522 31524257 PMC6691242

[B21] Ding S. Wang D. Zhou X. Chen L. Feng K. Xu X. (2022). Predicting Heart Cell Types by Using Transcriptome Profiles and a Machine Learning Method. Life 12, 228. 10.3390/life12020228 35207515 PMC8877019

[B22] Disease and Injury Incidence and Prevalence Collaborators (2017). Global, Regional, and National Incidence, Prevalence, and Years Lived with Disability for 328 Diseases and Injuries for 195 Countries, 1990-2016: a Systematic Analysis for the Global Burden of Disease Study 2016. Lancet 390, 1211–1259. 10.1016/S0140-6736(17)32154-2 28919117 PMC5605509

[B23] Donath M. Y. Størling J. Maedler K. Mandrup-Poulsen T. (2003). Inflammatory Mediators and Islet beta-cell Failure: a Link between Type 1 and Type 2 Diabetes. J. Mol. Med. 81, 455–470. 10.1007/s00109-003-0450-y 12879149

[B24] Draminski M. Rada-Iglesias A. Enroth S. Wadelius C. Koronacki J. Komorowski J. (2008). Monte Carlo Feature Selection for Supervised Classification. Bioinformatics 24, 110–117. 10.1093/bioinformatics/btm486 18048398

[B25] Eckardt K. Görgens S. W. Raschke S. Eckel J. (2014). Myokines in Insulin Resistance and Type 2 Diabetes. Diabetologia 57, 1087–1099. 10.1007/s00125-014-3224-x 24676645

[B26] Elango S. Venugopal S. Thangaraj K. Viswanadha V. P. (2014). Novel Mutations in ATPase 8, ND1 and ND5 Genes Associated with Peripheral Neuropathy of Diabetes. Diabetes Res. Clin. Pract. 103, e49–e52. 10.1016/j.diabres.2013.12.015 24456990

[B27] Ferrannini E. (2009). Insulin Resistance versus β-cell Dysfunction in the Pathogenesis of Type 2 Diabetes. Curr. Diab Rep. 9, 188–189. 10.1007/s11892-009-0031-8 19490819

[B28] Global Burden of Disease Cancer Collaboration Fitzmaurice C. Allen C. Barber R. M. Barregard L. Bhutta Z. A. (2017). Global, Regional, and National Cancer Incidence, Mortality, Years of Life Lost, Years Lived with Disability, and Disability-Adjusted Life-Years for 32 Cancer Groups, 1990 to 2015: A Systematic Analysis for the Global Burden of Disease Study. JAMA Oncol. 3, 524–548. 10.1001/jamaoncol.2016.5688 27918777 PMC6103527

[B29] Forst T. Anastassiadis E. Diessel S. Löffler A. Pfützner A. (2014). Effect of Linagliptin Compared with Glimepiride on Postprandial Glucose Metabolism, Islet Cell Function and Vascular Function Parameters in Patients with Type 2 Diabetes Mellitus Receiving Ongoing Metformin Treatment. Diabetes Metab. Res. Rev. 30, 582–589. 10.1002/dmrr.2525 24459063

[B30] Gambin T. Yuan B. Bi W. Liu P. Rosenfeld J. A. Coban-Akdemir Z. (2017). Identification of Novel Candidate Disease Genes from De Novo Exonic Copy Number Variants. Genome Med. 9, 83. 10.1186/s13073-017-0472-7 28934986 PMC5607840

[B31] Gao H. X. Regier E. E. Close K. L. (2016). International Diabetes Federation World Diabetes Congress 2015. J. Diabetes 8, 300. 10.1111/1753-0407.12377 26778343

[B32] Garufi A. Pistritto G. Baldari S. Toietta G. Cirone M. D’Orazi G. (2017). p53-Dependent PUMA to DRAM Antagonistic Interplay as a Key Molecular Switch in Cell-Fate Decision in Normal/high Glucose Conditions. J. Exp. Clin. Cancer Res. 36, 126. 10.1186/s13046-017-0596-z 28893313 PMC5594515

[B33] Golubnitschaja O. Moenkemann H. Trog D. B. Blom H. J. De Vriese A. S. (2006). Activation of Genes Inducing Cell-Cycle Arrest and of Increased DNA Repair in the Hearts of Rats with Early Streptozotocin-Induced Diabetes Mellitus. Med. Sci. Monit. 12, BR68–74. 16449944

[B34] Good A. L. Haemmerle M. W. Oguh A. U. Doliba N. M. Stoffers D. A. (2019). Metabolic Stress Activates an ERK/hnRNPK/DDX3X Pathway in Pancreatic β Cells. Mol. Metab. 26, 45–56. 10.1016/j.molmet.2019.05.009 31178390 PMC6667393

[B35] Guo X. Zhang Z. Gerstein M. B. Zheng D. (2009). Small RNAs Originated from Pseudogenes: Cis- or Trans-acting? PLoS Comput. Biol. 5, e1000449. 10.1371/journal.pcbi.1000449 19649160 PMC2708354

[B36] Houštek J. Hejzlarová K. Vrbacký M. Drahota Z. Landa V. Zídek V. (2012). Nonsynonymous Variants in Mt-Nd2, Mt-Nd4, and Mt-Nd5 Are Linked to Effects on Oxidative Phosphorylation and Insulin Sensitivity in Rat Conplastic Strains. Physiol. Genomics 44, 487–494. 10.1152/physiolgenomics.00156.2011 22414913 PMC3426424

[B37] Jia C. Zuo Y. Zou Q. (2018). O-GlcNAcPRED-II: an Integrated Classification Algorithm for Identifying O-GlcNAcylation Sites Based on Fuzzy Undersampling and a K-Means PCA Oversampling Technique. Bioinformatics 34, 2029–2036. 10.1093/bioinformatics/bty039 29420699

[B38] Jia Y. Zhao R. Chen L. (2020). Similarity-Based Machine Learning Model for Predicting the Metabolic Pathways of Compounds. IEEE Access 8, 130687–130696. 10.1109/access.2020.3009439

[B39] Johannes F. Widmer G. (1994). “Incremental Reduced Error Pruning,” in Proceedings of the Eleventh International Conference, Rutgers University, New Brunswick, NJ, July 10–July 13, 1994, 70–77. 10.1016/b978-1-55860-335-6.50017-9

[B40] Johnson D. S. (1974). Approximation Algorithms for Combinatorial Problems. J. Comput. Syst. Sci. 9, 256–278. 10.1016/s0022-0000(74)80044-9

[B41] Kahn S. E. (2003). The Relative Contributions of Insulin Resistance and Beta-Cell Dysfunction to the Pathophysiology of Type 2 Diabetes. Diabetologia 46, 3–19. 10.1007/s00125-002-1009-0 12637977

[B42] Kalyana-Sundaram S. Kumar-Sinha C. Shankar S. Robinson D. R. Wu Y.-M. Cao X. (2012). Expressed Pseudogenes in the Transcriptional Landscape of Human Cancers. Cell 149, 1622–1634. 10.1016/j.cell.2012.04.041 22726445 PMC3597446

[B43] Kandaswamy K. K. Chou K.-C. Martinetz T. Möller S. Suganthan P. N. Sridharan S. (2011). AFP-pred: A Random Forest Approach for Predicting Antifreeze Proteins from Sequence-Derived Properties. J. Theor. Biol. 270, 56–62. 10.1016/j.jtbi.2010.10.037 21056045

[B44] Kim J.-H. Choi S.-Y. Kang B.-H. Lee S.-M. Park H. S. Kang G.-Y. (2013). AMP-activated Protein Kinase Phosphorylates CtBP1 and Down-Regulates its Activity. Biochem. Biophysical Res. Commun. 431, 8–13. 10.1016/j.bbrc.2012.12.117 23291169

[B45] Kohavi R. (1995). “A Study of Cross-Validation and Bootstrap for Accuracy Estimation and Model Selection,” in International Joint Conference on Artificial Intelligence, Montreal Quebec Canada, August 20–August 25, 1995 (Lawrence Erlbaum Associates Ltd), 1137–1145.

[B46] Lawlor N. George J. Bolisetty M. Kursawe R. Sun L. Sivakamasundari V. (2017). Single-cell Transcriptomes Identify Human Islet Cell Signatures and Reveal Cell-type-specific Expression Changes in Type 2 Diabetes. Genome Res. 27, 208–222. 10.1101/gr.212720.116 27864352 PMC5287227

[B47] Lebovitz C. B. Robertson A. G. Goya R. Jones S. J. Morin R. D. Marra M. A. (2015). Cross-cancer Profiling of Molecular Alterations within the Human Autophagy Interaction Network. Autophagy 11, 1668–1687. 10.1080/15548627.2015.1067362 26208877 PMC4590660

[B48] Lee M.-S. (2014). Role of Islet β Cell Autophagy in the Pathogenesis of Diabetes. Trends Endocrinol. Metabolism 25, 620–627. 10.1016/j.tem.2014.08.005 25242548

[B49] Li X. Lu L. Lu L. Chen L. (2022). Identification of Protein Functions in Mouse with a Label Space Partition Method. Mbe 19, 3820–3842. 10.3934/mbe.2022176 35341276

[B50] Li Z. Wang D. Liao H. Zhang S. Guo W. Chen L. (2022). Exploring the Genomic Patterns in Human and Mouse Cerebellums via Single-Cell Sequencing and Machine Learning Method. Front. Genet. 13, 857851. 10.3389/fgene.2022.857851 35309141 PMC8930846

[B51] Liang H. Chen L. Zhao X. Zhang X. (2020). Prediction of Drug Side Effects with a Refined Negative Sample Selection Strategy. Comput. Math. Methods Med. 2020, 1573543. 10.1155/2020/1573543 32454877 PMC7232712

[B52] Liu H. Hu B. Chen L. Lu L. (2021). Identifying Protein Subcellular Location with Embedding Features Learned from Networks. Cp 18, 646–660. 10.2174/1570164617999201124142950

[B53] Liu H. Setiono R. (1998). Incremental Feature Selection. Appl. Intell. 9, 217–230. 10.1023/a:1008363719778

[B54] Liu J.-Y. Yao J. Li X.-M. Song Y.-C. Wang X.-Q. Li Y.-J. (2014). Pathogenic Role of lncRNA-MALAT1 in Endothelial Cell Dysfunction in Diabetes Mellitus. Cell Death Dis. 5, e1506. 10.1038/cddis.2014.466 25356875 PMC4649539

[B55] Lontchi-Yimagou E. Sobngwi E. Matsha T. E. Kengne A. P. (2013). Diabetes Mellitus and Inflammation. Curr. Diab Rep. 13, 435–444. 10.1007/s11892-013-0375-y 23494755

[B56] Lyons T. J. Basu A. (2012). Biomarkers in Diabetes: Hemoglobin A1c, Vascular and Tissue Markers. Transl. Res. 159, 303–312. 10.1016/j.trsl.2012.01.009 22424433 PMC3339236

[B57] Ma L. Zheng J. (2018). Single-cell Gene Expression Analysis Reveals β-cell Dysfunction and Deficit Mechanisms in Type 2 Diabetes. BMC Bioinforma. 19, 515. 10.1186/s12859-018-2519-1 PMC6311914 30598071

[B58] Mannino G. C. Sesti G. (2012). Individualized Therapy for Type 2 Diabetes: Clinical Implications Of Pharmacogenetic Data. Mol. Diagn Ther. 16, 285–302. 10.1007/s40291-012-0002-7 23018631

[B59] Marques Y. B. De Paiva Oliveira A. Ribeiro Vasconcelos A. T. Cerqueira F. R. (2016). Mirnacle: Machine Learning with SMOTE and Random Forest for Improving Selectivity in Pre-miRNA Ab Initio Prediction. BMC Bioinforma. 17, 474. 10.1186/s12859-016-1343-8 PMC5249014 28105918

[B60] Mathews C. E. Leiter E. H. Spirina O. Bykhovskaya Y. Gusdon A. M. Ringquist S. (2005). mt-Nd2 Allele of the ALR/Lt Mouse Confers Resistance against Both Chemically Induced and Autoimmune Diabetes. Diabetologia 48, 261–267. 10.1007/s00125-004-1644-8 15692809

[B61] Matthews B. W. (1975). Comparison of the Predicted and Observed Secondary Structure of T4 Phage Lysozyme. Biochimica Biophysica Acta (BBA) - Protein Struct. 405, 442–451. 10.1016/0005-2795(75)90109-9 1180967

[B62] Mella M. T. Kohari K. Jones R. Peña J. Ferrara L. Stone J. (2016). Mitochondrial Gene Expression Profiles Are Associated with Intrahepatic Cholestasis of Pregnancy. Placenta 45, 16–23. 10.1016/j.placenta.2016.07.002 27577705

[B63] Mirza A. H. Berthelsen C. H. Seemann S. E. Pan X. Frederiksen K. S. Vilien M. (2015). Transcriptomic Landscape of lncRNAs in Inflammatory Bowel Disease. Genome Med. 7, 39. 10.1186/s13073-015-0162-2 25991924 PMC4437449

[B64] Onesime M. Yang Z. Dai Q. (2021). Genomic Island Prediction via Chi-Square Test and Random Forest Algorithm. Comput. Math. Methods Med. 2021, 9969751. 10.1155/2021/9969751 34122622 PMC8169257

[B65] Pan X.-Y. Shen H.-B. (2009). Robust Prediction of B-Factor Profile from Sequence Using Two-Stage SVR Based on Random Forest Feature Selection. Ppl 16, 1447–1454. 10.2174/092986609789839250 20001907

[B66] Pan X. Li H. Zeng T. Li Z. Chen L. Huang T. (2021). Identification of Protein Subcellular Localization with Network and Functional Embeddings. Front. Genet. 11, 626500. 10.3389/fgene.2020.626500 33584818 PMC7873866

[B67] Pandey A. Chawla S. Guchhait P. (2015). Type-2 Diabetes: Current Understanding and Future Perspectives. IUBMB Life 67, 506–513. 10.1002/iub.1396 26177573

[B68] Poliseno L. Marranci A. Pandolfi P. P. (2015). Pseudogenes in Human Cancer. Front. Med. 2, 68. 10.3389/fmed.2015.00068 PMC4585173 26442270

[B69] Prentki M. Nolan C. J. (2006). Islet Cell Failure in Type 2 Diabetes. J. Clin. Investigation 116, 1802–1812. 10.1172/jci29103 PMC1483155 16823478

[B70] Pullen T. J. Rutter G. A. (2014). Roles of lncRNAs in Pancreatic Beta Cell Identity and Diabetes Susceptibility. Front. Genet. 5, 193. 10.3389/fgene.2014.00193 25071823 PMC4076741

[B71] Quinlan J. R. (1990). Learning Logical Definitions from Relations. Mach. Learn 5, 239–266. 10.1007/bf00117105

[B72] Segerstolpe Å. Palasantza A. Eliasson P. Andersson E.-M. Andréasson A.-C. Sun X. (2016). Single-Cell Transcriptome Profiling of Human Pancreatic Islets in Health and Type 2 Diabetes. Cell Metab. 24, 593–607. 10.1016/j.cmet.2016.08.020 27667667 PMC5069352

[B73] Siddiqui A. Madhu S. V. Sharma S. B. Desai N. G. (2015). Endocrine Stress Responses and Risk of Type 2 Diabetes Mellitus. Stress 18, 498–506. 10.3109/10253890.2015.1067677 26303379

[B74] Soto A. G. Cheruvu S. Bialo D. Quintos J. B. (2014). Refractory Diabetes Insipidus Leading to Diagnosis of Type 2 Diabetes Mellitus and Non-ketotic Hyperglycemia in an Adolescent Male. R. I. Med. J. (2013) 97, 34–35. 25083957

[B75] Stancakova A. Laakso M. (2016). Genetics of Type 2 Diabetes. Endocr. Dev. 31, 203–220. 10.2337/dc10-1013 26824439

[B76] Tang S. Chen L. (2022). iATC-NFMLP: Identifying Classes of Anatomical Therapeutic Chemicals Based on Drug Networks, Fingerprints and Multilayer Perceptron. Curr. Bioinforma. 17. 10.2174/1574893617666220318093000

[B77] Tao Z. Shi A. Zhao J. (2015). Epidemiological Perspectives of Diabetes. Cell Biochem. Biophys. 73, 181–185. 10.1007/s12013-015-0598-4 25711186

[B78] Tay Y. Rinn J. Pandolfi P. P. (2014). The Multilayered Complexity of ceRNA Crosstalk and Competition. Nature 505, 344–352. 10.1038/nature12986 24429633 PMC4113481

[B79] Taylor R. (2013). Type 2 Diabetes: Etiology And Reversibility. Diabetes Care 36, 1047–1055. 10.2337/dc12-1805 23520370 PMC3609491

[B80] Thorens B. (2014). Neural Regulation of Pancreatic Islet Cell Mass and Function. Diabetes Obes. Metab. 16 (Suppl. 1), 87–95. 10.1111/dom.12346 25200301

[B81] Torrell H. Montaña E. Abasolo N. Roig B. Gaviria A. M. Vilella E. (2013). Mitochondrial DNA (mtDNA) in Brain Samples from Patients with Major Psychiatric Disorders: Gene Expression Profiles, mtDNA Content and Presence of the mtDNA Common Deletion. Am. J. Med. Genet. 162, 213–223. 10.1002/ajmg.b.32134 23355257

[B82] Trujillo J. M. Nuffer W. (2014). GLP-1 Receptor Agonists for Type 2 Diabetes Mellitus: Recent Developments and Emerging Agents. Pharmacotherapy 34, 1174–1186. 10.1002/phar.1507 25382096

[B83] Tsai A. Cowan M. R. Johnson D. G. Brannon P. M. (1994). Regulation of Pancreatic Amylase and Lipase Gene Expression by Diet and Insulin in Diabetic Rats. Am. J. Physiology-Gastrointestinal Liver Physiology 267, G575–G583. 10.1152/ajpgi.1994.267.4.g575 7524347

[B84] Tseng C. H. Chen C. J. Landolph J. R. Jr. (2012). Diabetes and Cancer: Epidemiological, Clinical, and Experimental Perspectives. Exp. Diabetes Res. 2012, 101802. 10.1155/2012/101802 23082075 PMC3469104

[B85] Tsuyuki S. Takabayashi M. Kawazu M. Kudo K. Watanabe A. Nagata Y. (2014). Detection ofWIPI1mRNA as an Indicator of Autophagosome Formation. Autophagy 10, 497–513. 10.4161/auto.27419 24384561 PMC4077887

[B86] Urbanová M. Mráz M. Ďurovcová V. Trachta P. Kloučková J. Kaválková P. (2017). The Effect of Very-Low-Calorie Diet on Mitochondrial Dysfunction in Subcutaneous Adipose Tissue and Peripheral Monocytes of Obese Subjects with Type 2 Diabetes Mellitus. Physiol. Res. 66, 811–822. 10.33549/physiolres.933469 28730835

[B87] Walker W. E. Kurscheid S. Joshi S. Lopez C. A. Goh G. Choi M. (2015). Increased Levels of Macrophage Inflammatory Proteins Result in Resistance to R5-Tropic HIV-1 in a Subset of Elite Controllers. J. Virol. 89, 5502–5514. 10.1128/jvi.00118-15 25740989 PMC4442529

[B88] Wang Y. Xu Y. Yang Z. Liu X. Dai Q. (2021). Using Recursive Feature Selection with Random Forest to Improve Protein Structural Class Prediction for Low-Similarity Sequences. Comput. Math. Methods Med. 2021, 5529389. 10.1155/2021/5529389 34055035 PMC8123985

[B89] Wei L. Luan S. Nagai L. A. E. Su R. Zou Q. (2018). Exploring Sequence-Based Features for the Improved Prediction of DNA N4-Methylcytosine Sites in Multiple Species. Bioinformatics 35, 1326–1333. 10.1093/bioinformatics/bty824 30239627

[B90] Westermark G. T. Westermark P. (2008). Importance of Aggregated Islet Amyloid Polypeptide for the Progressive Beta-Cell Failure in Type 2 Diabetes and in Transplanted Human Islets. Exp. Diabetes Res. 2008, 528354. 10.1155/2008/528354 19343195 PMC2663347

[B91] Wu Z. Chen L. (2022). Similarity-based Method with Multiple-Feature Sampling for Predicting Drug Side Effects. Comput. Math. Methods Med. 2022, 1–13. 10.1155/2022/9547317 PMC8993545 35401786

[B92] Xin Y. Kim J. Okamoto H. Ni M. Wei Y. Adler C. (2016). RNA Sequencing of Single Human Islet Cells Reveals Type 2 Diabetes Genes. Cell Metab. 24, 608–615. 10.1016/j.cmet.2016.08.018 27667665

[B93] Yabe D. Seino Y. Fukushima M. Seino S. (2015). β Cell Dysfunction versus Insulin Resistance in the Pathogenesis of Type 2 Diabetes in East Asians. Curr. Diab Rep. 15, 602. 10.1007/s11892-015-0602-9 25944304 PMC4420838

[B94] Yang Y. Chen L. (2022). Identification of Drug-Disease Associations by Using Multiple Drug and Disease Networks. Cbio 17, 48–59. 10.2174/1574893616666210825115406

[B95] Zhang Y.-H. Li H. Zeng T. Chen L. Li Z. Huang T. (2021a). Identifying Transcriptomic Signatures and Rules for SARS-CoV-2 Infection. Front. Cell Dev. Biol. 8, 627302. 10.3389/fcell.2020.627302 33505977 PMC7829664

[B96] Zhang Y.-H. Li Z. Zeng T. Chen L. Li H. Huang T. (2021b). Detecting the Multiomics Signatures of Factor-specific Inflammatory Effects on Airway Smooth Muscles. Front. Genet. 11, 599970. 10.3389/fgene.2020.599970 33519902 PMC7838645

[B97] Zhang Y.-H. Zeng T. Chen L. Huang T. Cai Y.-D. (2021c). Determining Protein-Protein Functional Associations by Functional Rules Based on Gene Ontology and KEGG Pathway. Biochimica Biophysica Acta (BBA) - Proteins Proteomics 1869, 140621. 10.1016/j.bbapap.2021.140621 33561576

[B98] Zhao X. Chen L. Guo Z.-H. Liu T. (2019). Predicting Drug Side Effects with Compact Integration of Heterogeneous Networks. Cbio 14, 709–720. 10.2174/1574893614666190220114644

[B99] Zhao X. Chen L. Lu J. (2018). A Similarity-Based Method for Prediction of Drug Side Effects with Heterogeneous Information. Math. Biosci. 306, 136–144. 10.1016/j.mbs.2018.09.010 30296417

[B100] Zhou J.-P. Chen L. Wang T. Liu M. (2020b). iATC-FRAKEL: a Simple Multi-Label Web Server for Recognizing Anatomical Therapeutic Chemical Classes of Drugs with Their Fingerprints Only. Bioinformatics 36, 3568–3569. 10.1093/bioinformatics/btaa166 32154836

[B101] Zhou J. P. Chen L. Guo Z. H. (2020a). iATC-NRAKEL: An Efficient Multi-Label Classifier for Recognizing Anatomical Therapeutic Chemical Classes of Drugs. Bioinformatics 36, 1391–1396. 10.1093/bioinformatics/btz757 31593226

[B102] Zhou X. Hao Q. Liao J. Zhang Q. Lu H. (2013). Ribosomal Protein S14 Unties the MDM2-P53 Loop upon Ribosomal Stress. Oncogene 32, 388–396. 10.1038/onc.2012.63 22391559 PMC3736832

[B103] Zhu Y. Hu B. Chen L. Dai Q. (2021). iMPTCE-Hnetwork: A Multilabel Classifier for Identifying Metabolic Pathway Types of Chemicals and Enzymes with a Heterogeneous Network. Comput. Math. Methods Med. 2021, 6683051. 10.1155/2021/6683051 33488764 PMC7803417

[B104] Zick Y. (2001). Insulin Resistance: a Phosphorylation-Based Uncoupling of Insulin Signaling. Trends Cell Biol. 11, 437–441. 10.1016/s0962-8924(01)81297-6 11684411

